# Lack of Association between Oxytocin Receptor (OXTR) Gene Polymorphisms and Alexithymia: Evidence from Patients with Obsessive-Compulsive Disorder

**DOI:** 10.1371/journal.pone.0143168

**Published:** 2015-11-23

**Authors:** Min Jung Koh, Wonji Kim, Jee In Kang, Kee Namkoong, Se Joo Kim

**Affiliations:** 1 Department of Psychiatry, Bundang Jesaeng Hospital, Seongnam Gyeonggi, Republic of Korea; 2 Department of Psychiatry, Graduate school, Yonsei University, Seoul, Republic of Korea; 3 Interdisciplinary Program of Bioinformatics, Seoul National University, Seoul, Republic of Korea; 4 Department of Psychiatry and Institute of Behavioral Science in Medicine, Yonsei University College of Medicine, Seoul, Republic of Korea; NIMH, NIH, UNITED STATES

## Abstract

Oxytocin receptor gene single nucleotide polymorphisms have been associated with structural and functional alterations in brain regions, which involve social-emotional processing. Therefore, oxytocin receptor gene polymorphisms may contribute to individual differences in alexithymia, which is considered to be a dysfunction of emotional processing. The aim of this study was to evaluate the association between oxytocin receptor gene single nucleotide polymorphisms or haplotypes and alexithymia in patients with obsessive-compulsive disorder. We recruited 355 patients with obsessive-compulsive disorder (234 men, 121 women). Alexithymia was measured by using the Toronto Alexithymia Scale. We performed single-marker and haplotype association analyses with eight single nucleotide polymorphisms (rs237885, rs237887, rs2268490, rs4686301, rs2254298, rs13316193, rs53576, and rs2268498) in the oxytocin receptor gene. There were no significant associations between any of the eight single nucleotide polymorphism of the oxytocin receptor gene and alexithymia. In addition, a six-locus haplotype block (rs237885-rs237887-rs2268490-rs4686301-rs2254298-rs13316193) was not significantly associated with alexithymia. These findings suggest that genetic variations in the oxytocin receptor gene may not explain a significant part of alexithymia in patients with obsessive-compulsive disorder.

## Introduction

Alexithymia is characterized by difficulties in recognizing and effectively expressing one's own feelings [1]. It is difficult for highly alexithymic individuals to distinguish or appreciate the emotions of others [2], and this trait results in an unempathic and ineffective emotional response [3]. These individuals feel less distressed on seeing others in pain, and such blunted emotions can be translated into impaired prosociality [4]. Effective emotional processing, which is known to be impaired in alexithymia [5], is a key component for successful social behavior.

Large-scale twin studies reported that genetic factors could account for 30–42% of the individual differences in alexithymia [6, 7]. Early twin studies reported that the heritabilities of empathy, altruism, and nurturance ranged between 56–72%, and the effect of the shared environment was negligible [8, 9]. These findings suggest that there are some genetic influences on various human traits related to emotional processing, including alexithymia and other prosocial behaviors.

Oxytocin (OT) is a neuropeptide synthesized primarily in the paraventricular and supraoptic nuclei of the hypothalamus and plays a role as both a neurotransmitter and a neuromodulator. It is an important regulator of complex social behavior and emotional states, such as empathy, attachment, trust, social cognition, and emotional regulation[10–12] There is growing evidence suggesting a role of OT in the pathophysiology of several psychiatric conditions showing deficits in social functioning such as autism, mood disorder, schizophrenia, and so forth [13]. Intranasal administration of OT has been found to increase trust in unfamiliar persons, enhance facial affect recognition in autism [14], and improve the ability of affective “mind-reading” [15]. A recent study showed that oxytocin increased individuals’ willingness to share emotions [16]. Interestingly, the effect of OT on recognition of complex emotions is particularly pronounced in higher alexithymic individuals [17]. Therefore, OT may be associated with core characteristics of alexithymia such as decreased emotional recognition, expression, and emotional sharing [18].

The effects of OT are modulated by the expression and function of oxytocin receptors (OXTR). Several single nucleotide polymorphisms (SNPs) on the OXTR gene have been documented in association with various aspects of social-affective behavior as well as psychopathology [19–21]. OXTR SNPs have some influence on structural and functional changes in several brain regions involved in processing social-emotional information such as the prefrontal cortex, anterior cingulate cortex, amygdala, and hypothalamus [22], all of which are also important in the pathophysiology of alexithymia [23]. From this evidence, it is possible to speculate that the genetic variations of OXTR may influence individuals’ alexithymic traits.

With regard to obsessive-compulsive disorder (OCD), there have been several studies on the implications of alexithymia on OCD. Alexithymia is prevalent in OCD [24] and associated with poor insight [25], early age at onset, higher anxiety, and sexual/religious obsessions [26]. Studying the effect of the OXTR gene on alexithymia in OCD has several advantages. First, many of the previous genetic studies on alexithymia recruited participants from the normal population. This has limited such studies because of narrow variability in the alexithymic scores, which in turn reduced their power to detect differences. Therefore, it would be more fruitful to use samples with larger variability regarding alexithymia. Hence, we sought to investigate the association between OXTR genetic variants and alexithymia in patients with OCD, who supposedly have more variability in alexithymic traits than controls. Second, alexithymia is a candidate endophenotype for OCD because it is heritable [6, 7] and shares the core characteristics of OCD. Thus, elucidating the relationship between the OXTR gene and alexithymia may help identify predisposing genes for OCD.

## Materials and Methods

### Subjects

We recruited 355 patients (234 men, 121 women) from the OCD clinic at Severance Hospital, Yonsei University Health System (a tertiary referral hospital in Korea) from August 2006 to May 2015. All participants met the criteria for OCD, as determined by the Structured Clinical Interview for the DSM-IV (SCID) [27] assessed by a trained psychiatrist (S.J. KIM). This group was composed of patients in different stages of the illness and with different degrees of severity. All patients were taking psychotropic medications (mainly selective serotonin reuptake inhibitors and/or low-dose benzodiazepines). All participants were Korean and gave written informed consent prior to the beginning of this study. The study protocol was approved by the Institutional Review Board of Severance hospital, Yonsei University Health System.

### Assessment of alexithymia

The degree of alexithymia was measured with the Toronto Alexithymia Scale (TAS-20), which is a 20-item self-report scale with a five-point Likert-type scale[28]. The TAS-20 comprises three subdimensions: 1) difficulty in identifying feelings (DIF, seven items), 2) difficulty in describing feelings (DDF, five items) and 3) externally oriented thinking (EOT, eight items). The TAS-20 was previously proven to be valid and reliable [28] and was validated for the Korean population [29]. All participants completed the Korean version of the 20-item Toronto Alexithymia Scale [30].

### Measures of clinical symptoms

The clinical symptoms and the severity of OCD symptoms were evaluated by using the Yale-Brown obsessive-compulsive scale (Y-BOCS) [31]. The Y-BOCS is a reliable and valid 10-item scale administered by a clinician and used to assess the severity of obsessions and compulsions. Levels of depressive symptoms were assessed by using the Montgomery–Åsperg Depression Rating Scale (MADRS) [32], which is a well-known scale widely used by trained psychiatrists.

### SNP selection and Genotyping

**Fig 1:**
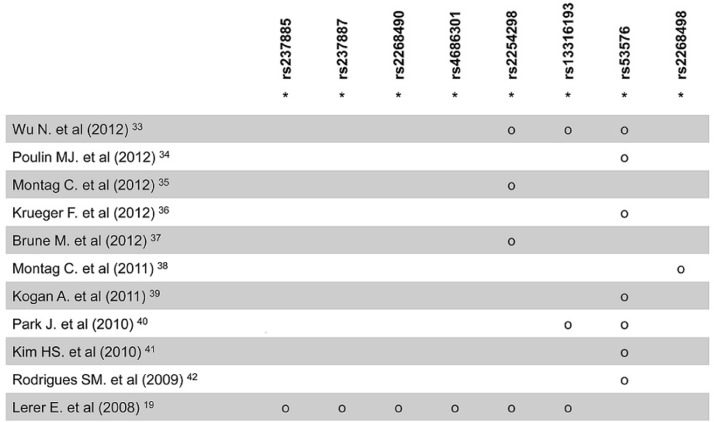
Selection of OXTR single-nucleotide polymorphism (SNP). Circle denote SNPs significantly associated with prosocial behavior in single-marker analyses of previous studies, with asterisks representing SNPs included the current association study.

First, we selected OXTR SNPs which showed significant association with empathy, prosocial behavior, sociability, emotionality, social cognition, and social deficit by using a PubMed search of previous studies [19, 33–42]. Nine SNPs were selected considering reported minor allele frequencies (MAF) greater than 0.1 from the 1000 Genomes Project database, JPT sample (June 2010 release). Among them, rs2268491 was excluded because it was in complete linkage disequilibrium (LD; D′ = 1, r^2^ = 0.97) with rs2254298 in our sample. Therefore, eight SNPs with an r^2^ threshold < 0.8 in ‘pair-wise tagging only’ mode using the ‘Tagger’ program in Haploview [43] were included in the final statistical analysis (Table 1; Fig 1). Peripheral blood samples were obtained from each subject, and genomic DNA was extracted from the leukocytes. The SNaPshot assay was performed according to the manufacturer’s instructions (ABI PRISM SNaPShot Multiplex kit, Foster City, CA, USA).

**Table 1:** Characteristics of SNP markers on the OXTR gene.

SNP No.	rs No.	Location	Statistical Tests for HWE			Allele	MAF
			χ^2^	df	p value		
1	rs237885	8795543	0.090	1	0.764	A>C	0.267
2	rs237887	8797042	0.159	1	0.690	C>T	0.428
3	rs2268490	8797085	0.006	1	0.936	G>A	0.450
4	rs4686301	8798586	0.095	1	0.758	G>A	0.193
5	rs2254298	8802228	0.017	1	0.898	C>T	0.279
6	rs13316193	8802743	0.563	1	0.453	A>G	0.168
7	rs53576	8804371	0.585	1	0.445	T>C	0.360
8	rs2268498	8812411	2.044	1	0.153	A>G	0.294

HWE, Hardy-Weinberg equilibrium; MAF, minor allele frequency; SNP, single nucleotide polymorphism.

rs No. indicates SNP identification in the dbSNP site of NCBI.

### Statistical analyses

Hardy-Weinberg equilibrium values of the SNPs were tested using *χ* ^*2*^ tests. Generalized estimating equations (GEE) were used to analyze the relationship between total or three subdimension scores of TAS-20 and SNPs, adjusting for age, total Y-BOCS, and MADRS scores. An additive model was applied by coding genotypes as 0, 1, or 2, depending on carrier status of the minor allele.

**Fig 2:**
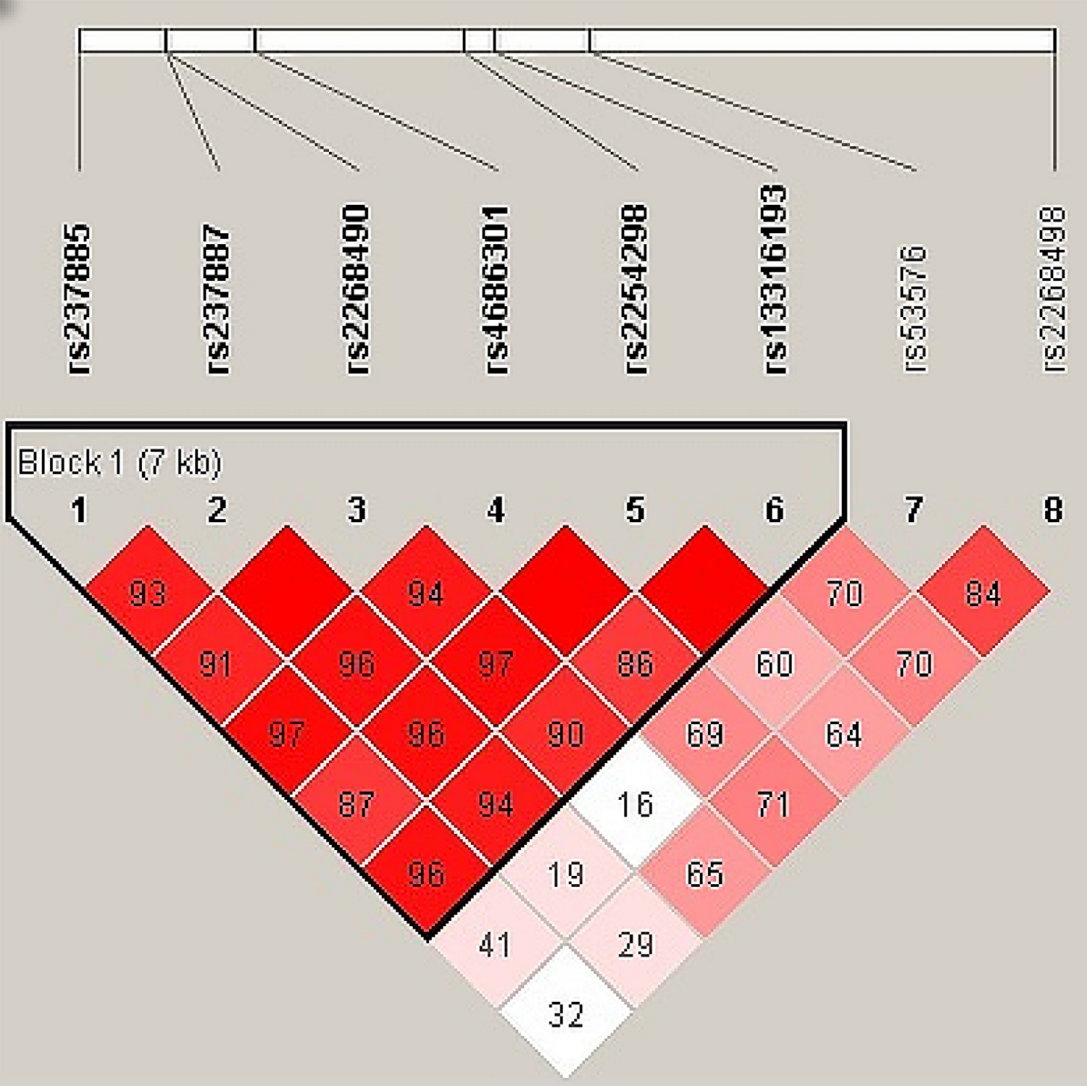
Linkage disequilibrium (LD) structure of the single-nucleotide polymorphisms and a haplotype block analyzed in the current study (Block1:SNP1-SNP6).

Haploview v4.0 (http://www.broadinstitute.org/haploview/haploview) [43] was used to estimate the pairwise LD of SNP markers. The default confidence interval algorithm of the Haploview program identified a single haplotype block consisting of SNP1 (rs237885), SNP2 (rs237887), SNP3 (rs2268490), SNP4 (rs4686301), SNP5 (rs2254298), and SNP6 (rs13316193) from our data (Fig 2). The associations between OXTR gene haplotypes and the TAS-20 total scores and scores of the three subdimensions were examined using the “haplo.score” function of the program ‘haplo.stats’ (http://cran.r-project.org/src/contrib/Descriptions/haplo.stats.html) controlling for age, total Y-BOCS and MADRS scores. Permutation tests (n = 10,000) were performed to estimate the global significance of the results for all haplotypes analyzed and to validate the expectation-maximization values.

## Results

Demographic and clinical characteristics of the subjects are presented in Table 2. The mean age of subjects was 30.48 ± 11.13 years old. The mean age of onset of obsessive-compulsive symptoms was 18.82 ± 9.21. The mean scores of Y-BOCS, and MADRS were 22.16 ± 8.70, and 19.07 ± 8.99, respectively. Because the patients’ total scores and some subdimension scores on the TAS-20 were significantly correlated with the total Y-BOCS and MADRS scores, and age, although the size of the correlation between the TAS-20 scores (total and subdimension scores) and the Y-BOCS or age were negligible (absolute r = 0.13–0.19, data not present), we used these factors as covariates in subsequent analyses to control for their potential effects on alexithymia.

**Table 2:** Demographic and clinical characteristics of subjects.

Variables	N = 355	Mean (SD) / n (%)
Sex	Men	234 (65.92)
Age (years)		30.48 (11.13)
Onset Age (years)		18.82 (9.21)
Education (years)		13.15 (2.87)
Y-BOCS total score		22.16 (8.70)
MADRS total score		19.07 (8.99)
TAS-20 score	Total	55.59 (11.63)
	DIF	19.63 (6.78)
	DDF	15.33 (4.46)
	EOT	20.63 (3.90)
Comorbidity	Depression	97 (27.32)
	Other anxiety disorders	51 (14.37)
	Other psychiatric conditions	10 (2.82)

SD, standard deviation; Y-BOCS, Yale-Brown Obsessive-Compulsive Scale; MADRS, Montgomery–Åsperg depression rating scale; TAS-20, Toronto Alexithymia Scale; DIF = Difficulty identifying feelings; DDF = Difficulty describing feelings; EOT = Externally oriented thinking.

We used GEE approaches to examine the effect of the OXTR gene polymorphisms on the total or subdimension scores of the TAS-20 for each of eight SNPs. There were no significant associations found between any of the OXTR SNPs and alexithymia. In addition, a six-locus haplotype block (rs237885-rs237887-rs2268490-rs4686301-rs2254298-rs13316193) also was not significantly associated with the severity of alexithymia. The results are shown in Tables 3, 4 and 5.

**Table 3:** The association between the OXTR functional SNPs and total TAS-20 scores divided by genotypes.

SNP	Genotype (n)	Total TAS scores	Regression			
			beta	Std. error	Z	*p* value
rs237885	AA (188)	56.12 (12.20)	-1.500	0.928	-1.616	0.107
	AC (140)	55.37 (10.73)				
	CC (24)	52.50 (11.96)				
rs237887	CC (114)	55.94 (11.98)	-0.475	0.833	-0.570	0.569
	CT (177)	55.39 (11.88)				
	TT (63)	55.49 (10.45)				
rs2268490	GG (106)	55.51 (10.08)	-0.049	0.822	-0.060	0.953
	AG (175)	56.09 (12.33)				
	AA (71)	55.14 (11.78)				
rs4686301	GG (231)	56.15 (12.06)	-1.756	1.017	-1.727	0.085
	GA (108)	54.32 (10.13)				
	AA (14)	54.07 (12.19)				
rs2254298	CC (183)	55.58 (11.15)	-0.555	0.905	-0.614	0.540
	CT (143)	55.70 (11.82)				
	TT (27)	54.19 (13.13)				
rs13316193	AA (244)	55.98 (11.91)	-0.878	1.114	-0.788	0.431
	AG (103)	54.17 (10.63)				
	GG (8)	61.75 (13.70)				
rs53576	TT (148)	55.79 (11.85)	-0.184	0.827	-0.222	0.824
	CT (156)	54.89 (11.23)				
	CC (49)	56.88 (11.65)				
rs2268498	AA (181)	55.55 (12.06)	0.062	0.861	0.072	0.943
	AG (135)	55.59 (10.55)				
	GG (36)	54.56 (12.83)				

Data are listed as mean (SD). TAS-20, Toronto Alexithymia Scale-20. Statistical significance was evaluated by GEE tests. Age, total Y-BOCS and MADRS score were used as covariates. All tests are two-tailed.

**Table 4:** The association between the OXTR functional SNPs and TAS-20 subdimension scores divided by genotypes.

SNP	Genotype (n)	TAS subdimension scores			GEE			
		DIF	DDF	EOT	beta	Std. error	Z	*p* value
rs237885	AA (188)	19.95(6.90)	15.44(4.57)	20.73(3.97)	-0.454	0.277	-1.642	0.101
	AC (140)	19.38(6.48)	15.54(4.38)	20.45(3.80)				
	CC (24)	18.58(7.66)	13.25(3.78)	20.67(4.04)				
rs237887	CC (114)	19.85(6.83)	15.38(4.46)	20.71(4.05)	-0.146	0.240	-0.610	0.542
	CT (177)	19.40(6.88)	15.34(4.70)	20.65(3.95)				
	TT (63)	19.95(6.55)	15.17(3.77)	20.37(3.50)				
rs2268490	GG (106)	19.43(6.30)	15.53(3.94)	20.55(3.77)	-0.064	0.238	-0.269	0.788
	AG (175)	19.79(7.02)	15.51(4.72)	20.79(4.09)				
	AA (71)	19.90(6.82)	14.85(4.40)	20.39(3.69)				
rs4686301	GG (231)	20.14(6.99)	15.37(4.56)	20.63(3.87)	-0.476	0.295	-1.611	0.107
	GA (108)	18.57(6.14)	15.37(4.31)	20.38(3.74)				
	AA (14)	18.64(7.24)	13.93(3.10)	21.50(4.43)				
rs2254298	CC (183)	19.46(6.75)	15.61(4.17)	20.51(3.98)	-0.169	0.280	-0.602	0.547
	CT (143)	19.87(6.53)	15.16(4.62)	20.67(3.85)				
	TT (27)	19.15(7.95)	14.22(5.30)	20.81(3.59)				
rs13316193	AA (244)	19.91(6.99)	15.33(4.58)	20.75(3.81)	-0.294	0.341	-0.862	0.389
	AG (103)	18.63(6.15)	15.33(4.27)	20.21(4.00)				
	GG (8)	24.13(6.27)	15.38(3.46)	22.25(4.71)				
rs53576	TT (148)	19.74(7.09)	15.25(4.69)	20.80(3.94)	-0.016	0.250	-0.063	0.950
	CT (156)	19.55(6.56)	15.15(4.11)	20.19(3.73)				
	CC (49)	19.33(6.42)	16.08(4.76)	21.47(4.06)				
rs2268498	AA (181)	19.73(6.77)	15.20(4.60)	20.62(3.88)	0.035	0.283	0.125	0.902
	AG (135)	19.53(6.81)	15.50(4.10)	20.56(3.92)				
	GG (36)	18.81(6.56)	15.17(5.05)	20.58(3.95)				

Data are listed as mean (SD). TAS-20, Toronto Alexithymia Scale-20; DIF, difficulties in identifying feelings; DDF, difficulties in describing feelings; EOT, externally oriented thinking. Statistical significance was evaluated by GEE tests. Age, total Y-BOCS and MADRS score were used as covariates. All tests are two-tailed.

**Table 5:** Results of haplotype-based quantitative trait-association analysis between TAS-20 scores and six SNP markers in the OXTR gene.

Haplotype	SNP 1-2-3-4-5-6	Hap_Frequency	TAS-20 scores (global-stat, df, *p* value, global sim. *p* value)											
			DIF			DDF			EOT			Total		
			(7.10, 9, 0.62, 0.64)			(11.83, 9, 0.22, 0.22)			(8.54, 9, 0.48, 0.49)			(8.50, 9, 0.48, 0.49)		
			Hap_score	*p*	sim. *p*	Hap_score	*p*	sim. *p*	Hap_score	*p*	sim. *p*	Hap_score	*p*	sim. *p*
1	A-C-A-G-T-A	0.27	0.12	0.91	0.91	-1.52	0.13	0.13	1.00	0.32	0.32	-0.15	0.88	0.88
2	A-C-A-G-C-A	0.18	0.68	0.50	0.49	0.23	0.82	0.82	-1.34	0.18	0.18	-0.03	0.98	0.98
3	A-T-G-G-C-A	0.16	0.72	0.47	0.47	0.78	0.44	0.45	0.11	0.91	0.91	0.75	0.46	0.46
4	C-T-G-A-C-G	0.15	-1.29	0.20	0.20	-0.95	0.34	0.34	-0.83	0.41	0.42	-1.40	0.16	0.17
5	A-C-G-G-C-A	0.12	-0.22	0.83	0.82	2.53	0.01	0.01	1.39	0.16	0.17	1.38	0.17	0.17
6	C-T-G-G-C-A	0.05	1.05	0.30	0.29	-0.67	0.50	0.50	0.01	0.99	0.99	0.33	0.74	0.74
7	C-T-G-A-C-A	0.04	-1.31	0.19	0.19	-0.37	0.71	0.72	0.50	0.62	0.62	-0.69	0.49	0.50
8	C-T-G-G-C-G	0.01	1.20	0.23	0.24	1.57	0.12	0.12	0.18	0.85	0.85	1.37	0.17	0.17
9	A-C-A-G-C-G	0.006	-0.60	0.55	0.55	-0.80	0.42	0.43	-1.34	0.18	0.18	-1.14	0.25	0.26

TAS-20, Toronto Alexithymia Scale-20; DIF, difficulties in identifying feelings; DDF, difficulties in describing feelings; EOT, externally oriented thinking; sim. *p* value, *p*-value based on permutations. Age, total Y-BOCS and MADRS score were adjusted. Statistical significance was evaluated by quantitative trait analysis using R version 3.3.1.

## Discussion

Initially, we hypothesized that OXTR variants might account for the individual differences of alexithymic traits seen in OCD patients. However, contrary to our initial hypothesis, there were no differences of alexithymic traits according to alleles or genotypes of eight SNPs within OXTR. Haplotype analyses also did not show any association between various haplotypes of OXTR and alexithymia. Alexithymic individuals show impaired emotional experience, deficits of emotional interpretation and emotional face recognition, and lack of empathy [44] and are associated with various psychiatric conditions including autistic disorders and psychopathy [45, 46]. Although there have been no reports which directly investigated the influence of OXTR genetic variants on alexithymic traits, there are a number of studies of the association between OXTR polymorphisms and those various alexithymia-related traits (e.g., empathy, emotional facial recognition, and so forth) and psychiatric disorders (e.g., autism spectrum disorders, psychopathy, and so forth).

Recently, Laursen et al. found that subjects with the CC genotype at OXTR rs2268498 and AA genotype at OXTR rs53576 showed higher empathic accuracy [47]. Meanwhile, Uzefovsky et al. reported that the A allele of OXTR rs53576 predicted lower emotional empathy [48]. Melcher et al. found that T allele carriers of OXTR rs2268498 showed more accurate facial emotional recognition skill [49]. In addition, subjects with the A allele of OXTR rs2254298 showed deficient deactivation of the dorsal anterior cingulate gyrus during an emotional face matching task [50]. The SNP rs13316193 C allele of the OXTR gene has been associated with empathy [33], whereas the T allele has been linked to decreased expression of oxytocin receptors in the brain, depressive mood, and greater risk for autism spectrum disorder [19]. One recent meta-analysis study found significant association between autism spectrum disorder and the rs7632287, rs237887, rs2268491, and rs2254298 SNPs of OXTR [51]. Psychopathy has a higher incidence of alexithymic traits, and both psychopathy and alexithymia have common core features of a lack of empathy, insight, and introspection [52]. Several neuroimaging studies suggested that the genetic variants of OXTR modulate the activities of limbic circuits including the amygdala, the hypothalamus, and the cingulate gyrus [53], which are also associated with alexithymia [23].

However, not all of the studies consistently reported a positive association between OXTR variants and alexithymia-related traits. Nyffeler et al. did not find any associations between single SNPs of OXTR (rs2301261, rs53576, rs2144298, or rs2268494) and autism [54]. Tansey et al. reported no association between 18 SNPs of OXTR and samples of autistic individuals from Ireland, Portugal, and the United Kingdom [55]. Also, a meta-analyses study consisting of a large number of participants (N ≥ 17000 for rs53576 and N ≥ for rs2254298) failed to support the impact of these two OXTR gene variants on five domains of human functioning including biology, personality, social behavior, psychopathology, and autism [56].

However, the resulting lack of association in our study should be interpreted with caution because there are several factors, which are required to be considered. First, the ethnic and cultural backgrounds might influence the results of our study. In fact, several inconclusive results about autism have been reported from different ethnic and cultural backgrounds across studies. In Japanese [57] and Chinese [58] populations, the rs2254398 A allele of OXTR has been reported to be linked to autism. However, in Caucasian autism trios, the rs2254395 G allele of OXTR was overtransmitted to probands with autistic disorders [59]. In addition, as mentioned earlier, one study of a Caucasian sample found no influence of various SNPs of the OXTR gene on autism [55], although they did not include the rs2254398. We did not control the potential effects of undetected population stratification. Notwithstanding, the Korean population is considered to be much more ethnically and culturally homogenous than other populations, due to its distinct language and culture. Although the Koreans are assumed to be relatively free from stratification [60], there is still potential risk of biased results from undetected population stratification. Second, the participants in this study were all OCD patients, and the disease status of the sample might influence the results. For example, in schizophrenic patients, the A allele carriers of the rs2254298 SNP of OXTR had higher empathic concern than non-A allele carriers, whereas in healthy controls, this difference was not found [35]. Therefore, although patients with OCD tend to be highly alexithymic with a relatively large variability, which can increase statistical power, the results cannot be generalized to a non-clinical sample or to other psychiatric conditions. Third, we did not consider gene and environment interaction effects. However, there has been evidence suggesting an OXTR gene by environment interaction on various human traits [56] (e.g., emotional dysregulation and attachment style) or psychiatric diseases [61] (e.g., depression). Lastly, while our sample size was relatively larger than those in previous studies examining associations between OXTR SNPs and alexithymia-related conditions, our power is still limited.

When we simulated the power of our study (5,000 trial runs) using the JPT + CHB panel of a 1,000 genome database and phenotypic variance-covariance matrix estimated from this study, the power of the sample size in this study was only 0.342, and was sufficient for detecting only an effect size larger than 0.9, as its regards to an association between rs237885 (the lowest GEE p-value in this study) and three subdimension scores of TAS-20. However, the effect size of rs237885 in this study was only 0.454 (standardized regression coefficient, β), suggesting the possibility of type II errors. Therefore, to confirm our reported findings, a study with a much larger number of samples with stronger statistical power is needed.

Therefore, it would be necessary to evaluate the effects of OXTR variants on alexithymia with particular consideration towards those confounding factors and limitations.

## Supporting Information

S1 Table Dataset of all subjects used in this study. (PDF)
