# Supplementary material for: Lack of Association between Oxytocin Receptor (OXTR) Gene Polymorphisms and Alexithymia: Evidence from Patients with Obsessive-Compulsive Disorder
Source: PLoS One. 2015 Nov 23;10(11):e0143168. doi: 10.1371/journal.pone.0143168 (PMC4658073; doi:10.1371/journal.pone.0143168)
Supplement: S1 Table — (PDF) [file pone.0143168.s001.pdf]

S1 Table. Dataset of all subjects used in this study.

| Sub. | Sex | Age | Onset | Education | SNP1 | SNP2 | SNP3 | SNP4 | SNP5 | SNP6 | SNP7 | SNP8 | TAS subdimension | | | Total | Total | Total |
|---|---|---|---|---|---|---|---|---|---|---|---|---|---|---|---|---|---|---|
| | | | age | year | rs237885 | rs237887 | rs2268490 | rs4686301 | rs2254298 | rs13316193 | rs53576 | rs2268498 | DIF | DDF | EOT | TAS | Y-BOCS | MADRS |
| 1 | 1 | 48 | 22 | 18 | AC | CT | GA | GA | CT | GA | CT | AA | 17 | 11 | 14 | 42 | 21 | 7 |
| 2 | 1 | 39 | 35 | 16 | AA | CT | GG | GG | CC | AA | TT | GG | 21 | 15 | 22 | 58 | 18 | 29 |
| 3 | 1 | 23 | 9 | 9 | AC | TT | GG | GG | CC | AA | TT | GG | 23 | 22 | 21 | 66 | 35 | 19 |
| 4 | 1 | 27 | 16 | 12 | AC | CT | GA | GA | CT | AA | CT | AA | 19 | 13 | 12 | 44 | 30 | 26 |
| 5 | 1 | 56 | 18 | 18 | AA | CC | GA | GG | CC | AA | CT | GA | 11 | 14 | 24 | 49 | 14 | 19 |
| 6 | 1 | 22 | 9 | 12 | AC | CT | GA | | CT | GA | CT | AA | 32 | 25 | 33 | 90 | 28 | 33 |
| 7 | 1 | 29 | 14 | 12 | CC | TT | GG | AA | CC | GA | CC | AA | 20 | 14 | 19 | 53 | 37 | 23 |
| 8 | 1 | 20 | 16 | 12 | AC | CT | GA | GA | CC | AA | CT | GA | 17 | 13 | 21 | 51 | 13 | 4 |
| 9 | 1 | 20 | 15 | 12 | AC | CT | GA | GA | CC | AA | CC | AA | 18 | 19 | 17 | 54 | 28 | 27 |
| 10 | 1 | 21 | 16 | 9 | AA | CT | GA | GG | CT | AA | TT | GG | 12 | 11 | 23 | 46 | 28 | 8 |
| 11 | 2 | 41 | 32 | 12 | AA | CC | AA | GG | CT | AA | TT | GA | 15 | 15 | 18 | 48 | 29 | 17 |
| 12 | 2 | 22 | 18 | 12 | AA | TT | GG | GG | CC | AA | CT | GA | 15 | 15 | 20 | 50 | 37 | 35 |
| 13 | 1 | 34 | 11 | 16 | AA | CC | AA | GG | CT | AA | CC | AA | 21 | 18 | 22 | 61 | 22 | 19 |
| 14 | 2 | 23 | 16 | 9 | AA | CT | GA | GG | CC | AA | CT | GA | 7 | 13 | 20 | 40 | 13 | 7 |
| 15 | 1 | 29 | 11 | 12 | AA | CC | AA | GG | CC | AA | CC | AA | 20 | 16 | 23 | 59 | 21 | 25 |
| 16 | 1 | 32 | 15 | 16 | AA | CC | AA | GG | TT | AA | TT | AA | 31 | 22 | 21 | 74 | 21 | 19 |
| 17 | 2 | 34 | 19 | 13 | AC | TT | GG | GG | CC | AA | TT | GG | 18 | 12 | 20 | 50 | 38 | 34 |
| 18 | 1 | 33 | 19 | 16 | AC | CT | GA | GA | CT | GA | TT | AA | 17 | 17 | 20 | 54 | 16 | 9 |
| 19 | 2 | 32 | 17 | 18 | AA | TT | GG | GG | CC | AA | TT | GG | 14 | 7 | 16 | 37 | 28 | 15 |
| 20 | 2 | 30 | 30 | 17 | AC | CT | GA | GA | CC | GA | CT | AA | 21 | 17 | 22 | 60 | 31 | 26 |
| 21 | 1 | 29 | 18 | 9 | CC | TT | GG | AA | CC | GA | CC | AA | 13 | 18 | 24 | 55 | 18 | 19 |
| 22 | 1 | 25 | 19 | 16 | AC | CT | GA | GA | CC | GA | CC | AA | 18 | 19 | 23 | 60 | 18 | 20 |
| 23 | 1 | 26 | 13 | 12 | AC | CT | GG | GA | CC | GA | CC | AA | 16 | 16 | 25 | 57 | 24 | 21 |
| 24 | 2 | 20 | 11 | 12 | CC | CT | GA | GG | CT | AA | TT | GA | 21 | 13 | 17 | 51 | 22 | 23 |
| 25 | 2 | 56 | 20 | 6 | AA | CC | AA | GG | CC | AA | TT | AA | 25 | 18 | 23 | 66 | 29 | 35 |
| 26 | 1 | 27 | 10 | 9 | AC | TT | GG | GA | CC | GA | TT | GA | 21 | 15 | 23 | 59 | 18 | 19 |
| 27 | 1 | 23 | 12 | 12 | CC | TT | GG | AA | CC | GG | CC | AA | 30 | 19 | 28 | 77 | 30 | 38 |

| | | | | | | | | | | | | | | | | | | |
|---|---|---|---|---|---|---|---|---|---|---|---|---|---|---|---|---|---|---|
| 28 | 2 | 35 | 25 | 12 | AC | TT | GG | GA | CC | GA | CT | GA | 18 | 16 | 12 | 46 | 29 | 12 |
| 29 | 2 | 35 | 8 | 14 | AC | CT | GA | GA | CC | AA | CT | AA | 18 | 10 | 23 | 51 | 29 | 19 |
| 30 | 2 | 43 | 43 | 6 | AA | CC | GA | GG | CT | AA | TT | GA | 17 | 17 | 24 | 58 | 24 | 16 |
| 31 | 1 | 23 | 19 | 12 | AC | CT | GA | GA | CC | GA | CC | GA | 12 | 7 | 21 | 40 | 27 | 33 |
| 32 | 2 | 25 | 14 | 16 | AA | CT | GA | GG | CT | AA | TT | AA | 24 | 10 | 16 | 50 | 24 | 19 |
| 33 | 1 | 25 | 18 | 9 | AA | CT | GA | GG | CC | AA | CT | GA | 9 | 6 | 17 | 32 | 26 | 19 |
| 34 | 1 | 59 | 40 | 12 | AC | CT | GG | GA | CC | GA | CT | AA | 7 | 9 | 22 | 38 | 15 | 7 |
| 35 | 1 | 40 | 18 | 16 | AA | CT | GG | GG | CC | AA | TT | GA | 14 | 13 | 22 | 49 | 14 | 9 |
| 36 | 1 | 22 | 12 | 12 | AA | CC | GG | GG | CC | AA | CT | AA | 13 | 16 | 24 | 53 | 30 | 11 |
| 37 | 1 | 19 | 15 | 12 | AC | CT | GA | GA | CT | GA | TT | AA | 19 | 12 | 20 | 51 | 20 | 19 |
| 38 | 2 | 32 | 8 | 16 | AA | CC | GA | GG | CT | AA | TT | GA | 31 | 16 | 14 | 61 | 0 | 19 |
| 39 | 2 | 47 | 33 | 6 | AA | CC | GA | GG | CT | AA | TT | GA | 21 | 17 | 35 | 73 | 31 | 16 |
| 40 | 1 | 18 | 13 | 12 | AA | CT | GG | GG | CC | AA | CT | GA | 23 | 19 | 17 | 59 | 21 | 9 |
| 41 | 2 | 28 | 25 | 18 | AA | CT | GA | GG | CT | AA | TT | GA | 23 | 15 | 17 | 55 | 32 | 31 |
| 42 | 1 | 21 | 15 | 16 | AC | CT | GA | GG | CC | AA | CT | GA | 35 | 19 | 26 | 80 | 18 | 10 |
| 43 | 1 | 19 | 15 | 12 | AC | CT | | GA | CT | AA | CT | AA | 14 | 15 | 21 | 50 | 30 | 18 |
| 44 | 1 | 28 | 20 | 9 | AC | CT | GA | GA | CC | GA | CC | AA | 23 | 16 | 23 | 62 | 29 | 30 |
| 45 | 2 | 54 | 51 | 16 | AA | CT | GA | GG | CT | AA | TT | GA | 13 | 13 | 23 | 49 | 24 | 5 |
| 46 | 1 | 28 | 12 | 12 | AA | CC | AA | GG | CT | AA | CT | AA | 22 | 16 | 20 | 58 | 31 | 23 |
| 47 | 2 | 32 | 28 | 9 | AC | CT | GA | GA | CT | GA | TT | GA | 20 | 16 | 25 | 61 | 0 | 19 |
| 48 | 1 | 22 | 17 | 12 | AC | CT | GG | GG | CC | AA | CT | GA | 27 | 20 | 24 | 71 | 25 | 19 |
| 49 | 1 | 19 | 15 | 12 | AC | CT | GA | GA | CC | GA | CC | AA | 9 | 10 | 22 | 41 | 30 | 21 |
| 50 | 1 | 24 | 14 | 12 | AA | CC | GA | GG | CT | AA | TT | GA | 27 | 20 | 23 | 70 | 33 | 34 |
| 51 | 2 | 57 | 25 | 12 | AA | CC | GG | GG | CC | AA | TT | GG | 11 | 14 | 25 | 50 | 0 | 19 |
| 52 | 2 | 39 | 26 | 9 | AA | TT | GG | GG | CC | AA | TT | GG | 17 | 16 | 28 | 61 | 11 | 2 |
| 53 | 1 | 60 | 56 | 12 | AC | CT | GG | GA | CC | GA | CT | GA | 20 | 19 | 22 | 61 | 17 | 22 |
| 54 | 1 | 27 | 8 | 12 | AC | CT | GG | GG | CC | AA | CT | GA | 20 | 12 | 17 | 49 | 32 | 19 |
| 55 | 2 | 44 | 42 | 16 | AA | CT | GA | GG | CC | AA | TT | GA | 11 | 14 | 25 | 50 | 21 | 19 |
| 56 | 1 | 19 | 19 | 13 | CC | TT | GG | AA | CC | GA | CC | AA | 11 | 12 | 26 | 49 | 23 | 10 |
| 57 | 1 | 21 | 15 | 9 | AC | CT | GA | GA | CT | GA | CT | GA | 20 | 18 | 20 | 58 | 27 | 20 |
| 58 | 1 | 21 | 17 | 13 | AC | CT | GA | GA | CC | GA | CT | GA | 11 | 13 | 24 | 48 | 39 | 27 |
| 59 | 2 | 47 | 19 | 13 | AC | CT | GA | GA | CC | GA | CC | AA | 19 | 18 | 23 | 60 | 26 | 11 |

| | | | | | | | | | | | | | | | | | | |
|---|---|---|---|---|---|---|---|---|---|---|---|---|---|---|---|---|---|---|
| 60 | 2 | 35 | 13 | 16 | AA | CC | AA | GG | CT | AA | CT | AA | 21 | 20 | 20 | 61 | 25 | 21 |
| 61 | 1 | 24 | 19 | 9 | CC | TT | GG | GA | CC | GA | TT | GA | 24 | 18 | 18 | 60 | 21 | 23 |
| 62 | 1 | 29 | 22 | 9 | AA | CT | GA | GG | CC | AA | CT | GA | 29 | 18 | 20 | 67 | 14 | 8 |
| 63 | 2 | 24 | 18 | 12 | AC | CT | GA | GG | CT | AA | TT | GA | 22 | 12 | 25 | 59 | 32 | 25 |
| 64 | 1 | 28 | 14 | 9 | | CC | AA | GG | CT | AA | TT | GA | 14 | 12 | 18 | 44 | 26 | 19 |
| 65 | 1 | 21 | 16 | 12 | AA | CC | AA | GG | TT | AA | CT | AA | 28 | 22 | 26 | 76 | 19 | 25 |
| 66 | 1 | 29 | 15 | 16 | AC | CT | GA | GA | CT | GA | CC | AA | 17 | 10 | 17 | 44 | 22 | 12 |
| 67 | 2 | 51 | 46 | 16 | AC | TT | GG | GA | CC | GA | CT | AA | 22 | 17 | 20 | 59 | 31 | 17 |
| 68 | 1 | 20 | 18 | 12 | AC | CT | GA | GA | CT | GA | CT | GA | 29 | 20 | 16 | 65 | 19 | 15 |
| 69 | 2 | 66 | 35 | 12 | AC | CT | GA | GA | CC | GA | CC | GA | 19 | 15 | 19 | 53 | 28 | 6 |
| 70 | 1 | 33 | 17 | 16 | AA | CT | GG | GG | CC | AA | TT | GA | 21 | 17 | 22 | 60 | 12 | 8 |
| 71 | 1 | 18 | 17 | 12 | AC | CT | GA | GA | CT | GA | TT | AA | 21 | 17 | 25 | 63 | 0 | 19 |
| 72 | 2 | 39 | 32 | 9 | AA | CC | GA | GG | CT | AA | TT | GG | 17 | 16 | 23 | 56 | 0 | 19 |
| 73 | 2 | 27 | 21 | 16 | AA | CT | GA | GG | CT | AA | CT | GA | 31 | 21 | 22 | 74 | 32 | 30 |
| 74 | 2 | 28 | 19 | 12 | AC | TT | GG | GG | CC | GA | CC | AA | 25 | 23 | 21 | 69 | 25 | 36 |
| 75 | 1 | 20 | 17 | 9 | AA | TT | GG | GG | CC | AA | TT | GG | 31 | 25 | 20 | 76 | 0 | 19 |
| 76 | 1 | 34 | 27 | 16 | | TT | GG | AA | CC | GG | CC | AA | 30 | 18 | 25 | 73 | 39 | 39 |
| 77 | 1 | 25 | 12 | 16 | AA | CT | GG | GG | CC | AA | TT | GG | 15 | 17 | 21 | 53 | 19 | 14 |
| 78 | 1 | 28 | 19 | 18 | AC | CT | GA | GA | CT | AA | CT | GA | 19 | 17 | 20 | 56 | 31 | 40 |
| 79 | 1 | 19 | 16 | 12 | AC | CT | GA | GA | CC | GA | CT | AA | 27 | 17 | 8 | 52 | 29 | 28 |
| 80 | 1 | 24 | 10 | 16 | AA | CC | GA | GG | CT | AA | CT | AA | 21 | 19 | 22 | 62 | 29 | 20 |
| 81 | 1 | 26 | 18 | 12 | AA | CC | GA | GG | CT | AA | TT | GA | 24 | 20 | 23 | 67 | 33 | 31 |
| 82 | 1 | 25 | 19 | 13 | AA | CC | AA | GG | CC | AA | CC | AA | 17 | 12 | 21 | 50 | 22 | 19 |
| 83 | 1 | 20 | 14 | 12 | CC | TT | GG | GA | CC | AA | TT | GG | 11 | 9 | 20 | 40 | 18 | 7 |
| 84 | 2 | 34 | 19 | 16 | AA | CC | AA | GG | CT | AA | TT | AA | 25 | 18 | 21 | 64 | 20 | 30 |
| 85 | 2 | 31 | 28 | 16 | AA | CT | GA | GG | CC | AA | CT | GA | 11 | 11 | 23 | 45 | 31 | 18 |
| 86 | 2 | 41 | 19 | 18 | AA | CC | AA | GG | CT | AA | CT | AA | 24 | 19 | 22 | 65 | 35 | 34 |
| 87 | 2 | 29 | 19 | 16 | AA | CC | AA | GG | CT | AA | CT | AA | 23 | 16 | 21 | 60 | 26 | 18 |
| 88 | 1 | 31 | 19 | 16 | AA | CC | AA | GG | TT | AA | CT | AA | 22 | 12 | 20 | 54 | 28 | 36 |
| 89 | 1 | 44 | 38 | 16 | AA | CC | AA | GG | TT | AA | CT | AA | 18 | 9 | 20 | 47 | 17 | 18 |
| 90 | 1 | 31 | 26 | 16 | AA | CT | GA | GG | CC | AA | TT | AA | 17 | 16 | 16 | 49 | 30 | 9 |
| 91 | 2 | 24 | 18 | 12 | AA | CC | AA | GG | CT | AA | TT | AA | 21 | 15 | 16 | 52 | 13 | 12 |

| | | | | | | | | | | | | | | | | | | |
|---|---|---|---|---|---|---|---|---|---|---|---|---|---|---|---|---|---|---|
| 92 | 1 | 53 | 47 | 12 | AA | CC | AA | GG | CT | AA | CT | AA | 30 | 21 | 27 | 78 | 30 | 40 |
| 93 | 1 | 36 | 13 | 16 | AA | CC | GA | GG | CC | AA | TT | AA | 12 | 11 | 16 | 39 | 12 | 6 |
| 94 | 1 | 54 | 40 | 9 | AC | CT | GA | GA | CT | GA | CT | AA | 10 | 9 | 20 | 39 | 7 | 12 |
| 95 | 1 | 24 | 9 | 12 | AC | CT | GA | GA | CT | GA | CC | AA | 20 | 17 | 25 | 62 | 27 | 33 |
| 96 | 1 | 16 | 10 | 9 | AA | CC | AA | GG | CT | AA | TT | AA | 27 | 16 | 16 | 59 | 12 | 20 |
| 97 | 2 | 42 | 21 | 14 | AA | CT | GA | GG | CT | AA | TT | GA | 21 | 21 | 23 | 65 | 25 | 30 |
| 98 | 1 | 27 | 4 | 16 | AA | CT | GA | GG | CC | AA | CT | GA | 23 | 15 | 9 | 47 | 24 | 25 |
| 99 | 1 | 32 | 27 | 16 | AA | CT | GA | GG | TT | AA | TT | AA | 13 | 11 | 17 | 41 | 28 | 31 |
| 100 | 1 | 20 | 18 | 12 | AA | CC | | GG | CT | AA | CT | AA | 11 | 9 | 18 | 38 | 24 | 7 |
| 101 | 1 | 24 | 12 | 9 | AA | CT | GG | GG | CC | AA | TT | GG | 18 | 13 | 16 | 47 | 25 | 19 |
| 102 | 1 | 48 | 20 | 18 | AA | CT | GA | GG | CC | AA | TT | GA | 30 | 20 | 22 | 72 | 0 | 19 |
| 103 | 2 | 29 | 22 | 16 | AA | CC | AA | GA | CT | AA | CT | AA | 14 | 13 | 15 | 42 | 23 | 22 |
| 104 | 1 | 39 | 16 | 16 | AA | CC | GA | GG | CT | AA | TT | AA | 19 | 12 | 20 | 51 | 6 | 3 |
| 105 | 1 | 26 | 16 | 16 | AC | CT | GA | GG | CC | AA | TT | | 23 | 19 | 24 | 66 | 18 | 20 |
| 106 | 2 | 23 | 20 | 9 | AC | CC | GA | GG | CC | AA | CT | AA | 31 | 19 | 16 | 66 | 24 | 12 |
| 107 | 1 | 26 | 25 | 16 | AC | CT | GA | GA | CC | GA | CT | AA | 22 | 20 | 22 | 64 | 16 | 13 |
| 108 | 2 | 30 | 19 | 18 | AA | CT | GA | GG | CC | AA | CT | GA | 31 | 17 | 21 | 69 | 25 | 22 |
| 109 | 2 | 35 | 17 | 16 | AA | CT | GA | GG | CC | AA | CT | GA | 30 | 18 | 21 | 69 | 25 | 21 |
| 110 | 2 | 23 | 21 | 12 | AC | CT | GA | GA | CT | AA | TT | AA | 27 | 20 | 22 | 69 | 21 | 25 |
| 111 | 1 | 24 | 12 | 12 | AA | CC | AA | GG | TT | AA | TT | AA | 11 | 13 | 22 | 46 | 12 | 17 |
| 112 | 2 | 36 | 20 | 12 | AC | CT | GA | GA | CT | GA | CT | AA | 28 | 21 | 24 | 73 | 27 | 30 |
| 113 | 1 | 21 | 10 | 12 | AA | CT | GA | GG | CT | AA | CT | GA | 20 | 16 | 21 | 57 | 22 | 38 |
| 114 | 1 | 18 | 17 | 9 | AC | CT | GG | GA | CC | GA | CT | GA | 17 | 15 | 22 | 54 | 26 | 15 |
| 115 | 2 | 39 | 9 | 16 | AA | CC | AA | GG | CT | AA | CT | AA | 25 | 16 | 23 | 64 | 29 | 39 |
| 116 | 2 | 39 | 22 | 16 | CC | TT | GG | GA | CC | GG | CT | AA | 28 | 20 | 28 | 76 | 22 | 27 |
| 117 | 1 | 23 | 18 | 9 | AC | TT | GG | GA | CC | GA | CT | GG | 22 | 19 | 24 | 65 | 16 | 8 |
| 118 | 2 | 27 | 16 | 12 | AC | TT | GG | GA | CC | GA | CT | GA | 10 | 12 | 18 | 40 | 14 | 18 |
| 119 | 1 | 52 | 27 | 14 | AA | CT | GA | GG | CT | AA | CT | AA | 20 | 14 | 24 | 58 | 20 | 20 |
| 120 | 1 | 30 | 19 | 13 | AC | TT | GG | GA | CC | GA | CC | AA | 15 | 16 | 21 | 52 | 34 | 19 |
| 121 | 1 | 24 | 16 | 12 | AA | CC | GA | GG | CT | AA | TT | GG | 27 | 24 | 24 | 75 | 22 | 19 |
| 122 | 1 | 53 | 19 | 16 | AC | CT | GG | GG | CC | AA | TT | GG | 19 | 13 | 24 | 56 | 20 | 12 |
| 123 | 1 | 21 | 8 | 12 | AC | CT | GA | GG | CC | AA | CT | GA | 28 | 16 | 19 | 63 | 39 | 33 |

| | | | | | | | | | | | | | | | | | | |
|---|---|---|---|---|---|---|---|---|---|---|---|---|---|---|---|---|---|---|
| 124 | 1 | 33 | 23 | 16 | AA | CC | AA | GG | CC | AA | CC | AA | 33 | 19 | 24 | 76 | 24 | 25 |
| 125 | 1 | 22 | 12 | 12 | AC | TT | GG | GG | CC | AA | TT | AA | 33 | 23 | 24 | 80 | 35 | 36 |
| 126 | 1 | 22 | 12 | 9 | AA | CC | GA | GG | CT | AA | TT | GA | 17 | 18 | 24 | 59 | 18 | 7 |
| 127 | 2 | 56 | 35 | 16 | AA | CC | | GG | TT | AA | TT | AA | 7 | 5 | 19 | 31 | 28 | 2 |
| 128 | 2 | 17 | 19 | 9 | AA | CC | AA | GG | CT | AA | TT | AA | 31 | 18 | 20 | 69 | 27 | 27 |
| 129 | 2 | 45 | 19 | 13 | AC | CT | GA | GA | CC | AA | CC | AA | 20 | 19 | 24 | 63 | 30 | 27 |
| 130 | 2 | 44 | 34 | 16 | AA | CT | GA | GG | | AA | | | 35 | 22 | 27 | 84 | 25 | 23 |
| 131 | 1 | 32 | 26 | 12 | AA | CC | AA | GG | CT | AA | TT | AA | 11 | 5 | 16 | 32 | 27 | 13 |
| 132 | 1 | 35 | 17 | 18 | CC | CT | GA | GG | CT | AA | CT | GA | 7 | 9 | 19 | 35 | 2 | 19 |
| 133 | 1 | 35 | 11 | 12 | AA | CC | AA | GG | TT | AA | CT | AA | 23 | 13 | 21 | 57 | 34 | 20 |
| 134 | 1 | 24 | 17 | 16 | AA | CC | GA | GG | CT | AA | TT | | 26 | 13 | 22 | 61 | 27 | 34 |
| 135 | 2 | 25 | 17 | 16 | AC | CT | GA | GA | CC | GA | CC | AA | 21 | 16 | 21 | 58 | 32 | 15 |
| 136 | 2 | 40 | 20 | 16 | AC | CT | GG | GA | CC | GA | CC | AA | 20 | 20 | 23 | 63 | 30 | 25 |
| 137 | 2 | 42 | 18 | 12 | AA | CC | AA | GG | TT | AA | CT | AA | 22 | 16 | 21 | 59 | 17 | 23 |
| 138 | 2 | 56 | 34 | 16 | AC | CT | GA | GA | CC | GA | CC | GA | 9 | 15 | 25 | 49 | 11 | 16 |
| 139 | 1 | 21 | 18 | 9 | AA | CC | AA | GG | TT | AA | TT | AA | 15 | 9 | 29 | 53 | 23 | 11 |
| 140 | 2 | 26 | 22 | 9 | AC | CT | GA | GA | CC | GA | CC | AA | 15 | 10 | 14 | 39 | 15 | 9 |
| 141 | 1 | 46 | 36 | 16 | AA | CT | GA | GG | CC | GA | CT | GA | 8 | 9 | 12 | 29 | 29 | 12 |
| 142 | 1 | 24 | 20 | 16 | AA | CC | AA | GG | CT | AA | TT | AA | 23 | 13 | 23 | 59 | 21 | 2 |
| 143 | 2 | 35 | 18 | 16 | AA | CC | GA | GG | CT | AA | TT | GA | 7 | 7 | 16 | 30 | 35 | 6 |
| 144 | 1 | 36 | 14 | 12 | AA | CT | GA | GG | CT | AA | TT | GA | 19 | 15 | 21 | 55 | 30 | 16 |
| 145 | 2 | 29 | 18 | 16 | CC | CT | GA | GA | CT | AA | CC | AA | 16 | 8 | 20 | 44 | 17 | 19 |
| 146 | 1 | 21 | 19 | 12 | AA | CT | GA | GG | CC | AA | TT | GA | 29 | 25 | 20 | 74 | 12 | 30 |
| 147 | 1 | 24 | 12 | 16 | AA | CT | GG | GG | CC | AA | CC | AA | 23 | 18 | 30 | 71 | 21 | 16 |
| 148 | 2 | 29 | 19 | 16 | AC | CT | GG | GA | CC | GA | CC | GA | 31 | 22 | 8 | 61 | 23 | 34 |
| 149 | 2 | 24 | 9 | 12 | AA | CT | GA | GG | CT | AA | TT | GA | 23 | 16 | 22 | 61 | 29 | 17 |
| 150 | 1 | 28 | 21 | 16 | AA | TT | GG | GG | CC | AA | CT | GA | 14 | 16 | 22 | 52 | 26 | 11 |
| 151 | 1 | 22 | 10 | 12 | CC | TT | GG | GA | CC | AA | CT | AA | 34 | 19 | 16 | 69 | 26 | 29 |
| 152 | 1 | 21 | 12 | 12 | AA | CT | GA | GG | | AA | CT | AA | 14 | 12 | 24 | 50 | 31 | 29 |
| 153 | 2 | 26 | 14 | 9 | AA | CT | GG | GG | CC | AA | TT | GA | 21 | 14 | 22 | 57 | 26 | 20 |
| 154 | 1 | 25 | 21 | 9 | AA | CT | GG | GG | CC | AA | TT | GA | 11 | 19 | 25 | 55 | 23 | 15 |
| 155 | 1 | 24 | 19 | 16 | | CC | GG | GG | CC | AA | TT | GA | 16 | 16 | 23 | 55 | 0 | 37 |

| | | | | | | | | | | | | | | | | | | |
|---|---|---|---|---|---|---|---|---|---|---|---|---|---|---|---|---|---|---|
| 156 | 2 | 37 | 28 | 14 | AC | CT | GA | GG | CC | AA | TT | GA | 8 | 8 | 26 | 42 | 36 | 18 |
| 157 | 1 | 26 | 15 | 9 | AC | CT | GA | GA | CT | GA | CT | AA | 26 | 14 | 27 | 67 | 30 | 24 |
| 158 | 1 | 20 | 8 | 12 | AA | CT | GA | GG | CT | AA | TT | GA | 29 | 11 | 17 | 57 | 29 | 18 |
| 159 | 1 | 42 | 6 | 16 | AC | TT | GG | GG | CC | AA | TT | GA | 35 | 14 | 12 | 61 | 21 | 19 |
| 160 | 1 | 30 | 19 | 16 | AC | CT | GA | GA | CC | GA | CT | AA | 13 | 16 | 20 | 49 | 19 | 19 |
| 161 | 1 | 23 | 20 | 12 | AC | CT | GA | GG | CC | AA | CT | AA | 11 | 8 | 19 | 38 | 32 | 24 |
| 162 | 1 | 26 | 18 | 16 | AC | CT | GA | GA | CT | GA | TT | GA | 28 | 18 | 13 | 59 | 22 | 19 |
| 163 | 2 | 21 | 14 | 12 | AC | CT | GA | GA | CT | GA | CT | AA | 16 | 12 | 24 | 52 | 30 | 25 |
| 164 | 1 | 24 | 10 | 12 | AA | CT | GG | GG | CC | AA | TT | GG | 23 | 18 | 24 | 65 | 12 | 15 |
| 165 | 1 | 18 | 16 | 12 | AA | CC | AA | GG | TT | AA | TT | GA | 14 | 12 | 24 | 50 | 10 | 23 |
| 166 | 1 | 19 | 16 | 6 | AA | CT | GA | GG | CT | AA | TT | GG | 12 | 10 | 16 | 38 | 12 | 19 |
| 167 | 2 | 58 | 27 | 9 | AC | CT | GG | GA | CC | GA | CT | AA | 19 | 17 | 23 | 59 | 28 | 10 |
| 168 | 1 | 34 | 15 | 16 | AA | CC | AA | GG | TT | AA | TT | AA | 13 | 7 | 18 | 38 | 19 | 19 |
| 169 | 1 | 37 | 18 | 16 | AC | CT | GA | GA | CT | GA | CT | AA | 16 | 16 | 20 | 52 | 22 | 19 |
| 170 | 1 | 20 | 13 | 13 | AA | CC | AA | GG | CC | AA | CT | GA | 18 | 9 | 16 | 43 | 20 | 17 |
| 171 | 1 | 19 | 19 | 12 | AA | CC | GA | GG | CT | AA | TT | GA | 19 | 18 | 30 | 67 | 30 | 14 |
| 172 | 1 | 20 | 8 | 12 | AA | CC | GA | GG | CC | AA | CC | AA | 32 | 25 | 25 | 82 | 38 | 35 |
| 173 | 1 | 20 | 16 | 12 | AA | CT | GA | GG | CT | AA | TT | AA | 28 | 15 | 23 | 66 | 30 | 25 |
| 174 | 1 | 25 | 13 | 12 | AA | CC | AA | GG | TT | AA | TT | AA | 11 | 9 | 21 | 41 | 20 | 4 |
| 175 | 2 | 26 | 10 | 16 | AC | CT | GA | GA | CT | GA | CT | AA | 11 | 5 | 12 | 28 | 35 | 17 |
| 176 | 2 | 31 | 17 | 16 | AA | CC | GA | GG | CT | AA | CT | GA | 10 | 11 | 15 | 36 | 0 | 19 |
| 177 | 1 | 40 | 12 | 16 | AC | TT | GG | GG | CC | AA | TT | GA | 23 | 15 | 18 | 56 | 0 | 19 |
| 178 | 1 | 63 | 35 | 16 | CC | TT | GG | AA | CC | GA | CC | AA | 12 | 11 | 12 | 35 | 30 | 18 |
| 179 | 1 | 23 | 4 | 16 | AA | CT | GA | GG | CC | AA | CT | GA | 23 | 20 | 27 | 70 | 18 | 18 |
| 180 | 1 | 28 | 13 | 9 | AA | CT | GG | GG | CC | AA | TT | GG | 28 | 22 | 20 | 70 | 24 | 13 |
| 181 | 2 | 20 | 13 | 9 | AC | CT | GG | GG | CC | GA | CT | AA | 21 | 13 | 15 | 49 | 27 | 10 |
| 182 | 1 | 33 | 18 | 12 | AC | CT | GA | GA | CT | GA | TT | GA | 18 | 17 | 20 | 55 | 22 | 2 |
| 183 | 1 | 23 | 13 | 12 | AA | CT | GA | GG | CT | AA | TT | GA | 34 | 23 | 19 | 76 | 19 | 42 |
| 184 | 1 | 42 | 20 | 9 | AA | CC | AA | GG | CT | AA | CT | AA | 19 | 16 | 22 | 57 | 18 | 3 |
| 185 | 2 | 40 | 19 | 18 | AA | CT | GG | GG | CC | AA | TT | GG | 27 | 11 | 18 | 56 | 24 | 31 |
| 186 | 2 | 36 | 15 | 14 | AA | CC | AA | GG | TT | AA | TT | AA | 23 | 16 | 19 | 58 | 15 | 4 |
| 187 | 1 | 20 | 13 | 9 | AC | TT | GG | GA | CC | GA | CT | GA | 18 | 20 | 17 | 55 | 20 | 15 |

| | | | | | | | | | | | | | | | | | | |
|---|---|---|---|---|---|---|---|---|---|---|---|---|---|---|---|---|---|---|
| 188 | 1 | 19 | 17 | 12 | AA | CT | GA | GG | CC | AA | TT | GG | 10 | 10 | 15 | 35 | 31 | 10 |
| 189 | 1 | 20 | 19 | 9 | AA | CT | GA | GG | CT | AA | TT | AA | 31 | 19 | 23 | 73 | 32 | 32 |
| 190 | 1 | 26 | 14 | 16 | AC | CT | GA | GG | CT | GA | TT | AA | 31 | 20 | 15 | 66 | 20 | 19 |
| 191 | 2 | 32 | 18 | 16 | AC | CT | GA | GA | CT | GA | CT | AA | 34 | 20 | 18 | 72 | 21 | 26 |
| 192 | 2 | 22 | 14 | 12 | AA | CC | GA | GG | CT | AA | CC | AA | 29 | 25 | 25 | 79 | 31 | 32 |
| 193 | 1 | 29 | 18 | 16 | AA | CT | GG | GG | CC | AA | CT | GA | 25 | 20 | 25 | 70 | 20 | 11 |
| 194 | 2 | 24 | 22 | 9 | AA | CC | AA | GG | CT | AA | TT | AA | 23 | 11 | 12 | 46 | 18 | 24 |
| 195 | 2 | 57 | 25 | 12 | AC | CT | GA | GA | CT | AA | CT | AA | 11 | 14 | 25 | 50 | 27 | 11 |
| 196 | 2 | 22 | 12 | 12 | AC | TT | GG | GA | CC | AA | TT | GG | 29 | 17 | 22 | 68 | 27 | 20 |
| 197 | 1 | 26 | 18 | 12 | AC | TT | GG | GA | CC | GA | CT | GA | 24 | 21 | 19 | 64 | 14 | 9 |
| 198 | 1 | 72 | 52 | 16 | AA | CC | AA | GG | CT | AA | CT | GA | 7 | 8 | 21 | 36 | 19 | 8 |
| 199 | 1 | 20 | 16 | 12 | AC | TT | GG | GA | CC | GA | CT | GA | 26 | 17 | 18 | 61 | 0 | 19 |
| 200 | 1 | 21 | 14 | 16 | AA | CT | GA | GG | CT | AA | CT | GA | 22 | 20 | 22 | 64 | 22 | 16 |
| 201 | 2 | 25 | 14 | 16 | AA | CC | AA | GG | TT | AA | TT | AA | 30 | 17 | 21 | 68 | 0 | 19 |
| 202 | 2 | 28 | 12 | 16 | AA | CC | GG | GG | CC | AA | TT | GG | 22 | 17 | 10 | 49 | 29 | 25 |
| 203 | 2 | 36 | 32 | 16 | AA | CT | GA | GG | CT | AA | CT | GA | 25 | 18 | 19 | 62 | 19 | 9 |
| 204 | 1 | 22 | 17 | 12 | AC | TT | GG | GA | CC | GA | TT | GA | 9 | 11 | 19 | 39 | 10 | 10 |
| 205 | 1 | 26 | 11 | 12 | AA | CT | GG | GG | CC | AA | CT | AA | 16 | 22 | 19 | 57 | 11 | 28 |
| 206 | 2 | 33 | 11 | 18 | CC | TT | GG | AA | CC | GG | CT | GA | 25 | 15 | 17 | 57 | 37 | 42 |
| 207 | 2 | 19 | 19 | 9 | AA | CT | GA | GG | CC | AA | CT | GA | 12 | 19 | 21 | 52 | 27 | 14 |
| 208 | 1 | 32 | 11 | 16 | AA | | GA | GG | CT | AA | TT | GA | 16 | 18 | 23 | 57 | 30 | 28 |
| 209 | 1 | 23 | 9 | 12 | AC | CT | GA | GA | CT | GA | CT | GA | 13 | 10 | 18 | 41 | 12 | 11 |
| 210 | 2 | 23 | 21 | 12 | AC | CT | GA | GA | CT | GA | CT | AA | 17 | 13 | 19 | 49 | 26 | 29 |
| 211 | 2 | 37 | 14 | 16 | AA | CC | AA | GG | TT | AA | CT | AA | 14 | 18 | 14 | 46 | 31 | 24 |
| 212 | 1 | 36 | 16 | 16 | AC | CT | GA | GG | CC | AA | TT | GA | 23 | 19 | 19 | 61 | 11 | 6 |
| 213 | 2 | 52 | 37 | 9 | AC | CT | GA | GA | CT | AA | TT | AA | 22 | 16 | 23 | 61 | 22 | 15 |
| 214 | 1 | 52 | 17 | 18 | AC | CT | GA | GG | CC | AA | CT | GA | 21 | 18 | 15 | 54 | 11 | 14 |
| 215 | 1 | 56 | 10 | 16 | AA | CC | AA | GG | CT | GA | TT | AA | 16 | 14 | 18 | 48 | 24 | 21 |
| 216 | 1 | 16 | 11 | 9 | AA | CC | GA | GG | CT | AA | CT | AA | 18 | 16 | 21 | 55 | 0 | 19 |
| 217 | 2 | 22 | 13 | 12 | CC | TT | GG | AA | CC | GA | CC | AA | 18 | 16 | 25 | 59 | 27 | 29 |
| 218 | 1 | 29 | 22 | 16 | AC | CT | GA | GG | CC | GA | CC | AA | 15 | 14 | 19 | 48 | 19 | 5 |
| 219 | 1 | 30 | 26 | 16 | AC | CT | GA | GA | CT | GA | CT | AA | 17 | 12 | 19 | 48 | 19 | 5 |

| | | | | | | | | | | | | | | | | | | |
|---|---|---|---|---|---|---|---|---|---|---|---|---|---|---|---|---|---|---|
| 220 | 2 | 33 | 18 | 16 | CC | CT | GA | GA | CT | AA | TT | GA | 9 | 6 | 25 | 40 | 27 | 9 |
| 221 | 2 | 36 | 19 | 13 | AC | CT | GG | GA | CC | GA | TT | GG | 7 | 9 | 19 | 35 | 23 | 20 |
| 222 | 1 | 31 | 27 | 16 | AA | CC | AA | GA | CT | AA | TT | AA | 12 | 21 | 18 | 51 | 24 | 19 |
| 223 | 1 | 28 | 22 | 16 | AC | TT | GG | GA | CC | GA | CT | GA | 25 | 16 | 18 | 59 | 16 | 19 |
| 224 | 1 | 36 | 12 | 16 | AA | CC | AA | GG | CT | AA | CT | AA | 26 | 19 | 20 | 65 | 33 | 27 |
| 225 | 2 | 20 | 19 | 12 | AA | CC | AA | | CC | AA | CT | GA | 17 | 12 | 21 | 50 | 29 | 8 |
| 226 | 1 | 21 | 19 | 12 | AC | CT | GA | GG | CT | AA | TT | GA | 29 | 25 | 20 | 74 | 11 | 14 |
| 227 | 1 | 57 | 18 | 12 | AA | CC | AA | GG | TT | AA | TT | GA | 29 | 15 | 21 | 65 | 33 | 26 |
| 228 | 1 | 53 | 25 | 16 | CC | TT | GG | AA | CC | GG | CC | AA | 11 | 10 | 16 | 37 | 30 | 11 |
| 229 | 2 | 37 | 33 | 16 | AA | CT | GA | GG | CC | AA | CT | AA | 12 | 7 | 23 | 42 | 25 | 7 |
| 230 | 2 | 21 | 14 | 12 | AA | CT | GA | GG | CT | AA | TT | GA | 14 | 9 | 15 | 38 | 28 | 20 |
| 231 | 2 | 29 | 16 | 16 | AA | CC | AA | GG | CC | AA | CT | AA | 10 | 10 | 12 | 32 | 13 | 7 |
| 232 | 1 | 41 | 17 | 16 | AC | CT | GG | GA | CC | GA | CT | GA | 20 | 11 | 23 | 54 | 34 | 31 |
| 233 | 1 | 19 | 18 | 12 | AA | CT | GA | GG | CT | AA | TT | GA | 12 | 13 | 22 | 47 | 20 | 16 |
| 234 | 2 | 30 | 14 | 16 | AA | CC | GA | GG | CT | AA | TT | GA | 24 | 18 | 21 | 63 | 26 | 18 |
| 235 | 1 | 18 | 6 | 9 | AC | CT | GA | GA | CC | GA | CT | AA | 18 | 11 | 15 | 44 | 32 | 39 |
| 236 | 1 | 35 | 19 | 16 | AA | CC | AA | GG | CT | AA | TT | GA | 15 | 17 | 23 | 55 | 21 | 21 |
| 237 | 1 | 25 | 18 | 12 | AA | CC | GA | GG | CT | AA | CT | AA | 18 | 10 | 12 | 40 | 21 | 7 |
| 238 | 2 | 25 | 15 | 16 | AC | TT | GG | GG | CC | GA | CT | GA | 21 | 19 | 24 | 64 | 30 | 13 |
| 239 | 1 | 19 | 14 | 12 | AA | CC | GA | GG | CC | AA | CT | GA | 32 | 18 | 24 | 74 | 32 | 14 |
| 240 | 1 | 25 | 6 | 16 | AA | CC | GG | GG | CC | AA | CT | GA | 23 | 19 | 20 | 62 | 29 | 28 |
| 241 | 1 | 26 | 11 | 16 | CC | TT | GG | GG | CC | AA | TT | AA | 20 | 12 | 18 | 50 | 13 | 19 |
| 242 | 1 | 17 | 10 | 9 | AA | CC | AA | GG | CT | AA | CT | AA | 22 | 16 | 22 | 60 | 30 | 6 |
| 243 | 2 | 21 | 20 | 12 | AA | CC | AA | GG | CT | AA | TT | AA | 7 | 8 | 19 | 34 | 24 | 22 |
| 244 | 1 | 24 | 15 | 9 | AA | CT | GA | GG | CT | AA | TT | GG | 14 | 9 | 14 | 37 | 10 | 19 |
| 245 | 2 | 26 | 24 | 16 | AC | CT | GA | GA | CT | GA | CT | AA | 10 | 8 | 19 | 37 | 0 | 19 |
| 246 | 1 | 19 | 12 | 12 | AC | CT | GG | GA | CC | AA | CC | AA | 8 | 6 | 20 | 34 | 17 | 5 |
| 247 | 1 | 21 | 18 | 12 | AA | CC | AA | GG | TT | AA | CT | AA | 17 | 11 | 21 | 49 | 13 | 14 |
| 248 | 1 | 29 | 19 | 9 | AA | CT | GG | GG | CC | AA | CT | GA | 25 | 17 | 18 | 60 | 18 | 21 |
| 249 | 1 | 20 | 19 | 9 | AA | CC | GA | GG | CT | AA | TT | GA | 22 | 18 | 24 | 64 | 27 | 29 |
| 250 | 1 | 26 | 12 | 12 | AA | CC | AA | GG | CC | AA | TT | AA | 29 | 16 | 14 | 59 | 27 | 35 |
| 251 | 2 | 43 | 39 | 16 | AA | CC | AA | GG | CT | AA | TT | AA | 21 | 14 | 21 | 56 | 27 | 11 |

| | | | | | | | | | | | | | | | | | | |
|---|---|---|---|---|---|---|---|---|---|---|---|---|---|---|---|---|---|---|
| 252 | 1 | 56 | 10 | 16 | AC | TT | GG | GA | CC | GA | CT | AA | 16 | 14 | 18 | 48 | 25 | 37 |
| 253 | 1 | 22 | 12 | 12 | AC | TT | GG | GA | CC | AA | CT | GA | 22 | 15 | 19 | 56 | 29 | 28 |
| 254 | 2 | 44 | 32 | 12 | AA | CC | AA | GG | CT | AA | TT | AA | 17 | 14 | 25 | 56 | 9 | 14 |
| 255 | 1 | 25 | 18 | 12 | AC | CT | GA | GA | CT | GA | CT | AA | 29 | 21 | 20 | 70 | 31 | 32 |
| 256 | 1 | 24 | 12 | 9 | CC | TT | GG | GA | CC | GA | CT | AA | 15 | 13 | 19 | 47 | 15 | 2 |
| 257 | 1 | 62 | 55 | 9 | AA | CT | GA | GG | CC | AA | TT | GA | 20 | 12 | 25 | 57 | 15 | 24 |
| 258 | 2 | 58 | 52 | 9 | AC | CT | GG | GA | CC | GA | CT | GA | 8 | 11 | 20 | 39 | 0 | 19 |
| 259 | 1 | 26 | 24 | 16 | CC | TT | GG | AA | CC | GA | CC | AA | 11 | 9 | 22 | 42 | 3 | 6 |
| 260 | 1 | 29 | 15 | 16 | AA | CC | AA | GG | TT | AA | TT | AA | 26 | 21 | 25 | 72 | 19 | 19 |
| 261 | 1 | 23 | 16 | 12 | AC | CT | GG | GA | CC | AA | CC | AA | 13 | 22 | 27 | 62 | 18 | 3 |
| 262 | 2 | 23 | 15 | 16 | AC | CT | GG | GG | CC | AA | CT | AA | 23 | 19 | 22 | 64 | 21 | 32 |
| 263 | 1 | 21 | 14 | 12 | CC | TT | GG | AA | CC | GG | CC | AA | 24 | 14 | 24 | 62 | 20 | 21 |
| 264 | 1 | 23 | 22 | 12 | AC | CT | GA | GG | CC | AA | TT | GA | 9 | 9 | 16 | 34 | 28 | 10 |
| 265 | 2 | 21 | 14 | 12 | AA | CC | GA | GG | CC | AA | CT | AA | 17 | 16 | 17 | 50 | 20 | 6 |
| 266 | 1 | 23 | 13 | 9 | AA | CT | GG | GG | CC | AA | CT | GA | 23 | 21 | 23 | 67 | 23 | 9 |
| 267 | 1 | 24 | 17 | 12 | AA | CC | GA | GG | CC | AA | CT | GA | 26 | 20 | 20 | 66 | 15 | 20 |
| 268 | 1 | 32 | 22 | 16 | AC | CT | GA | GA | CT | GA | CT | AA | 15 | 10 | 18 | 43 | 19 | 19 |
| 269 | 2 | 39 | 33 | 9 | AC | CT | GA | GG | CT | AA | TT | GG | 13 | 11 | 25 | 49 | 33 | 19 |
| 270 | 2 | 43 | 43 | 12 | AA | CC | GA | GG | CC | AA | CT | GA | 9 | 10 | 25 | 44 | 10 | 7 |
| 271 | 1 | 23 | 10 | 9 | AC | TT | GG | GA | CC | GA | CT | GA | 16 | 11 | 22 | 49 | 16 | 19 |
| 272 | 1 | 41 | 12 | 16 | AA | CT | GA | GG | CT | AA | CC | AA | 23 | 10 | 18 | 51 | 13 | 9 |
| 273 | 1 | 19 | 14 | 13 | AC | TT | GG | GA | CC | GA | CC | AA | 23 | 17 | 20 | 60 | 22 | 19 |
| 274 | 1 | 20 | 14 | 12 | AA | CC | GA | GG | CT | AA | CT | AA | 25 | 14 | 18 | 57 | 20 | 23 |
| 275 | 2 | 19 | 8 | 13 | AC | CT | GA | GA | CT | GA | CC | AA | 24 | 23 | 25 | 72 | 8 | 19 |
| 276 | 2 | 33 | 7 | 16 | AC | CT | GA | GG | CT | GA | CT | AA | 18 | 7 | 17 | 42 | 0 | 19 |
| 277 | 2 | 48 | 35 | 16 | CC | TT | GG | AA | CC | GA | CT | AA | 11 | 12 | 23 | 46 | 9 | 19 |
| 278 | 1 | 19 | 9 | 12 | AA | CC | AA | GG | CT | AA | CT | AA | 21 | 15 | 22 | 58 | 28 | 9 |
| 279 | 1 | 22 | 14 | 16 | AC | CT | GA | GA | CC | GA | CC | AA | 17 | 18 | 18 | 53 | 19 | 25 |
| 280 | 1 | 21 | 17 | 12 | AA | CT | GG | GG | CC | AA | | GA | 16 | 12 | 15 | 43 | 17 | 16 |
| 281 | 1 | 22 | 19 | 9 | AC | TT | GG | GA | CC | GA | CT | GA | 20 | 17 | 24 | 61 | 15 | 19 |
| 282 | 2 | 26 | 19 | 16 | AA | CC | AA | GG | CC | AA | TT | GA | 20 | 21 | 31 | 72 | 29 | 12 |
| 283 | 2 | 56 | 28 | 9 | AC | TT | GG | GG | CC | AA | TT | GG | 22 | 15 | 22 | 59 | 17 | 15 |

| | | | | | | | | | | | | | | | | | | |
|---|---|---|---|---|---|---|---|---|---|---|---|---|---|---|---|---|---|---|
| 284 | 1 | 22 | 10 | 9 | AA | CT | GA | GG | CT | AA | CT | AA | 18 | 16 | 21 | 55 | 24 | 19 |
| 285 | 1 | 20 | 18 | 12 | AC | CT | GA | GG | CC | AA | TT | GA | 26 | 17 | 18 | 61 | 33 | 11 |
| 286 | 1 | 17 | 13 | 14 | AA | CT | GA | GG | CT | AA | TT | GA | 21 | 15 | 17 | 53 | 20 | 19 |
| 287 | 1 | 30 | 13 | 9 | AA | CC | GA | GG | CT | AA | TT | GA | 25 | 24 | 23 | 72 | 34 | 41 |
| 288 | 1 | 30 | 13 | 9 | AC | CT | GG | GA | CC | AA | TT | GA | 25 | 24 | 23 | 72 | 34 | 41 |
| 289 | 1 | 34 | 6 | 16 | AA | CT | GA | GG | CT | AA | CT | GA | 25 | 12 | 21 | 58 | 19 | 19 |
| 290 | 1 | 26 | 19 | 13 | AC | CT | GA | GA | CC | GA | CC | GA | 14 | 16 | 22 | 52 | 25 | 28 |
| 291 | 1 | 48 | 11 | 12 | AC | TT | GG | GA | CC | GA | CT | GA | 21 | 12 | 22 | 55 | 31 | 25 |
| 292 | 1 | 34 | 12 | 18 | AC | CT | GA | GA | CT | AA | CT | AA | 26 | 19 | 25 | 70 | 29 | 4 |
| 293 | 1 | 19 | 18 | 9 | CC | TT | GG | GA | CC | GA | CT | AA | 30 | 14 | 20 | 64 | 29 | 14 |
| 294 | 1 | 21 | 13 | 12 | AA | CC | AA | GG | TT | AA | TT | AA | 32 | 24 | 25 | 81 | 32 | 37 |
| 295 | 1 | 44 | 29 | 16 | AA | CC | AA | GG | CT | AA | CT | AA | 18 | 16 | 22 | 56 | 20 | 8 |
| 296 | 1 | 26 | 23 | 16 | AC | TT | GG | GA | CC | GA | TT | GG | 16 | 15 | 18 | 49 | 20 | 16 |
| 297 | 2 | 67 | 65 | 6 | AC | TT | GG | GA | CC | AA | CT | AA | 13 | 14 | 22 | 49 | 12 | 9 |
| 298 | 2 | 33 | 29 | 16 | AC | TT | GG | GG | CC | AA | CT | AA | 11 | 12 | 24 | 47 | 0 | 19 |
| 299 | 2 | 19 | 8 | 12 | CC | TT | GG | AA | CC | GG | CC | AA | 25 | 15 | 21 | 61 | 32 | 30 |
| 300 | 1 | 22 | 16 | 16 | AC | TT | GG | GA | CC | GA | CT | AA | 20 | 21 | 21 | 62 | 21 | 25 |
| 301 | 1 | 40 | 24 | 16 | CC | TT | GG | AA | CC | GG | CC | AA | 20 | 12 | 19 | 51 | 36 | 19 |
| 302 | 2 | 33 | 17 | 16 | AA | CC | AA | GG | CT | AA | CT | AA | 17 | 11 | 20 | 48 | 23 | 10 |
| 303 | 1 | 47 | 26 | 16 | AC | CT | GA | GA | CT | GA | CT | AA | 11 | 13 | 26 | 50 | 27 | 33 |
| 304 | 1 | 28 | 25 | 9 | AC | TT | GG | GG | CT | AA | TT | AA | 7 | 10 | 19 | 36 | 24 | 19 |
| 305 | 1 | 22 | 15 | 9 | AA | CC | GA | GG | CC | AA | CT | GG | 16 | 19 | 20 | 55 | 31 | 22 |
| 306 | 1 | 27 | 15 | 16 | AA | CC | AA | GG | CT | AA | CT | AA | 13 | 15 | 16 | 44 | 23 | 9 |
| 307 | 2 | 23 | 15 | 16 | AA | CT | GA | GG | CT | AA | TT | GA | 20 | 15 | 19 | 54 | 20 | 21 |
| 308 | 1 | 22 | 18 | 12 | AA | CC | AA | GG | CT | AA | CT | AA | 13 | 5 | 15 | 33 | 26 | 27 |
| 309 | 1 | 27 | 19 | 12 | AA | CC | AA | GG | TT | AA | TT | AA | 32 | 24 | 15 | 71 | 21 | 21 |
| 310 | 1 | 52 | 58 | 9 | AA | CT | GA | GG | CT | AA | TT | GA | 12 | 18 | 24 | 54 | 3 | 6 |
| 311 | 1 | 31 | 18 | 16 | AC | CT | GA | GA | CC | GA | CT | AA | 19 | 21 | 23 | 63 | 15 | 19 |
| 312 | 1 | 22 | 16 | 12 | AC | CT | GA | GA | CC | GA | TT | GA | 20 | 18 | 18 | 56 | 8 | 1 |
| 313 | 2 | 26 | 8 | 12 | AC | CT | GA | GA | CC | GA | CT | AA | 18 | 17 | 25 | 60 | 21 | 18 |
| 314 | 1 | 42 | 19 | 9 | AA | CT | GA | GG | CT | AA | TT | GA | 9 | 8 | 24 | 41 | 12 | 30 |
| 315 | 2 | 35 | 19 | 12 | AC | CT | GA | GG | CT | AA | TT | AA | 22 | 18 | 21 | 61 | 29 | 38 |

| | | | | | | | | | | | | | | | | | |
|---|---|---|---|---|---|---|---|---|---|---|---|---|---|---|---|---|---|
| 316 | 1 | 23 | 21 | 12 | AA | CC | GA | GG | CT | AA | CT | AA | 24 | 18 | 23 | 65 | 32 | 17 |
| 317 | 2 | 55 | 30 | 12 | AC | CC | AA | GG | CT | GA | CT | AA | 30 | 21 | 22 | 73 | 21 | 30 |
| 318 | 1 | 22 | 9 | 9 | AA | CT | GA | GG | CC | AA | CT | GA | 26 | 19 | 23 | 68 | 19 | 18 |
| 319 | 1 | 20 | 10 | 12 | AA | CC | GA | GG | CT | AA | TT | GG | 18 | 16 | 20 | 54 | 24 | 8 |
| 320 | 1 | 29 | 15 | 9 | AA | CC | AA | GG | TT | AA | CT | AA | 19 | 17 | 16 | 52 | 26 | 19 |
| 321 | 1 | 33 | 13 | 18 | AA | CC | GA | GG | CC | AA | CT | GA | 25 | 13 | 17 | 55 | 21 | 19 |
| 322 | 1 | 37 | 14 | 9 | AC | CT | GA | GA | CT | GA | CT | AA | 8 | 9 | 24 | 41 | 26 | 13 |
| 323 | 1 | 34 | 14 | 16 | AC | CT | GA | GG | CC | AA | CT | AA | 27 | 19 | 16 | 62 | 24 | 30 |
| 324 | 2 | 39 | 24 | 16 | AA | CC | GA | GG | CT | AA | TT | GA | 23 | 17 | 23 | 63 | 17 | 19 |
| 325 | 1 | 54 | 27 | 16 | AA | CC | AA | GG | TT | AA | TT | AA | 7 | 12 | 20 | 39 | 6 | 19 |
| 326 | 1 | 24 | 17 | 12 | AC | CT | GA | GA | CT | GA | CC | AA | 30 | 24 | 17 | 71 | 15 | 23 |
| 327 | 1 | 25 | 14 | 16 | AC | CT | GA | GA | CT | GA | CT | AA | 23 | 22 | 20 | 65 | 30 | 10 |
| 328 | 1 | 28 | 25 | 16 | AA | CC | AA | GG | CT | GA | CT | AA | 22 | 13 | 24 | 59 | 26 | 10 |
| 329 | 2 | 22 | 18 | 12 | AA | CC | GA | GG | CC | AA | CT | GA | 15 | 14 | 19 | 48 | 21 | 18 |
| 330 | 1 | 23 | 7 | 12 | AA | CC | AA | GG | TT | AA | TT | AA | 17 | 11 | 19 | 47 | 29 | 21 |
| 331 | 1 | 23 | 19 | 16 | AA | CC | AA | GG | TT | AA | CT | AA | 23 | 19 | 19 | 61 | 30 | 28 |
| 332 | 1 | 35 | 23 | 16 | AA | CC | AA | GG | CC | AA | CT | AA | 11 | 12 | 20 | 43 | 23 | 6 |
| 333 | 1 | 20 | 14 | 12 | AC | CT | GA | GA | CC | GA | CC | AA | 22 | 20 | 24 | 66 | 27 | 11 |
| 334 | 2 | 37 | 19 | 13 | AC | TT | GG | GG | CC | AA | TT | GG | 18 | 12 | 16 | 46 | 22 | 19 |
| 335 | 2 | 26 | 7 | 16 | AA | CT | GA | GG | CC | AA | TT | AA | 32 | 25 | 26 | 83 | 28 | 29 |
| 336 | 2 | 19 | 6 | 12 | AC | CT | GA | GG | CT | AA | TT | GG | 9 | 10 | 24 | 43 | 23 | 18 |
| 337 | 1 | 36 | 14 | 16 | AA | CC | GA | GG | CT | AA | TT | GA | 12 | 20 | 26 | 58 | 20 | 21 |
| 338 | 1 | 51 | 45 | 9 | AC | CT | GA | GA | CC | GA | CC | GA | 15 | 21 | 19 | 55 | 14 | 21 |
| 339 | 1 | 33 | 22 | 12 | AA | CC | AA | GG | TT | AA | TT | AA | 7 | 9 | 28 | 44 | 16 | 5 |
| 340 | 2 | 23 | 12 | 16 | AA | CT | GG | GG | CC | AA | TT | GA | 21 | 17 | 16 | 54 | 26 | 18 |
| 341 | 2 | 32 | 30 | 16 | AC | TT | GG | GG | CC | GA | TT | GG | 23 | 18 | 18 | 59 | 24 | 16 |
| 342 | 1 | 19 | 16 | 9 | AA | CT | GA | GG | CT | AA | TT | GG | 29 | 25 | 26 | 80 | 20 | 31 |
| 343 | 1 | 41 | 19 | 18 | AC | CT | GG | GG | CC | AA | TT | AA | 12 | 15 | 21 | 48 | 21 | 16 |
| 344 | 1 | 19 | 16 | 12 | AA | CT | GA | GG | CT | AA | CT | GA | 18 | 14 | 19 | 51 | 9 | 12 |
| 345 | 2 | 41 | 19 | 16 | AC | TT | GG | GG | CC | AA | TT | GG | 19 | 14 | 22 | 55 | 22 | 11 |
| 346 | 1 | 21 | 20 | 12 | AA | CT | GA | GG | CT | AA | CT | AA | 20 | 10 | 21 | 51 | 12 | 13 |
| 347 | 1 | 22 | 15 | 12 | AA | CT | GA | GG | CC | AA | TT | GG | 32 | 25 | 27 | 84 | 35 | 19 |

| | | | | | | | | | | | | | | | | | | |
|---|---|---|---|---|---|---|---|---|---|---|---|---|---|---|---|---|---|---|
| 348 | 2 | 31 | 26 | 16 | AA | CC | AA | GG | CT | AA | CT | AA | 29 | 19 | 19 | 67 | 29 | 10 |
| 349 | 2 | 48 | 46 | 16 | AA | CC | AA | GG | CT | AA | CT | AA | 21 | 17 | 18 | 56 | 34 | 30 |
| 350 | 2 | 22 | 17 | 12 | AA | CT | GG | GG | CC | AA | TT | GG | 14 | 10 | 18 | 42 | 22 | 7 |
| 351 | 1 | 24 | 6 | 14 | AC | CT | GA | GG | CT | AA | TT | GA | 17 | 11 | 22 | 50 | 15 | 8 |
| 352 | 1 | 30 | 9 | 16 | AC | TT | GG | GA | CC | GA | CT | GA | 19 | 12 | 18 | 49 | 23 | 17 |
| 353 | 1 | 25 | 17 | 12 | AC | TT | GG | GA | CC | GA | TT | GA | 20 | 12 | 21 | 53 | 18 | 12 |
| 354 | 1 | 22 | 20 | 16 | AA | CC | AA | GG | TT | AA | TT | GA | 13 | 10 | 20 | 43 | 21 | 20 |
| 355 | 2 | 19 | 14 | 12 | AC | TT | GG | GG | CC | AA | TT | GA | 20 | 11 | 20 | 51 | 32 | 26 |
